# ESeroS-GS Protects Neuronal Cells from Oxidative Stress by Stabilizing Lysosomes

**DOI:** 10.3390/molecules21060637

**Published:** 2016-05-25

**Authors:** Na Yang, Qianqian Chen, Xiaolong He, Xingyu Zhao, Taotao Wei

**Affiliations:** 1 National Laboratory of Biomacromolecules, Institute of Biophysics, Chinese Academy of Sciences, Beijing 100101, China; nayang2007@126.com (N.Y.); chenqian0621@163.com (Q.C.); xiaolonghe@yeah.net (X.H.); pkuibpzxy@gmail.com (X.Z.); 2 University of Chinese Academy of Sciences, Beijing 100049, China

**Keywords:** ESeroS-GS, antioxidant, neuronal cell, cell death, oxidative stress, lysosome, Bid

## Abstract

γ-l-glutamyl-*S*-[2-[[[3,4-dihydro-2,5,7,8-tetramethyl-2-(4,8,12-trimethyltridecyl)-2*H*-1-benzopyran-6-yl]oxy]carbonyl]-3-[[2-(1*H*-indol-3-yl)ethyl]amino]-3-oxopropyl]-l-cysteinylglycine sodium salt (ESeroS-GS) is a water-soluble derivative of α-tocopherol (vitamin E). We reported previously that ESeroS-GS can act as an anti-inflammatory agent and can induce cell death in breast cancer cells. However, the potential antioxidant capacities of ESeroS-GS remain elusive. Here, we measured its scavenging effects on free radicals and evaluated its protective effects on neuronal cells against oxidative stress. The results indicated that ESeroS-GS effectively scavenged both 2,2’-azinobis(3-ethylbenzothiazoline)-6-sulfonate free radicals (ABTS^•+^) and 2,2-diphenyl-1-picrylhydrazyl (DPPH) free radicals, and attenuated H_2_O_2_-induced neuronal cell death. H_2_O_2_ treatment induced lysosomal membrane permeabilization rapidly, and caused the redistribution of lysosomal proteases, which were responsible for the neuronal cell death. ESeroS-GS abolished the interaction between tBid and the lysosomal membranes, blocked the translocation of tBid to the lysosomal membranes, decreased its oligomerization within the membrane circumstances, prevented the lysosomal membrane permeabilization, and thus attenuated the neuronal cell death. These data suggest that ESeroS-GS protected the neuronal cells from oxidative stress by stabilizing lysosomal membranes, and thus might act as a novel neuroprotector for neuronal diseases associated with oxidative stress.

## 1. Introduction

In our body, a number of biochemical reactions involve the generation of reactive oxygen species (ROS). The major sources of endogenous ROS are mitochondria. Leakage of electrons during oxidative phosphorylation causes, depending on the animal species and rate of respiration, the conversion of 0.25%–11% of the oxygen utilized to superoxide anion radicals and, ultimately, H_2_O_2_ [1]. Under certain pathological conditions, when ROS are not effectively eliminated by the antioxidant defense system, the dynamic balance between the generation and diminution of ROS is broken. Excessive ROS can attack lipids, carbohydrates, proteins and DNA, thus resulting in oxidative stress. Several lines of evidence link ROS to the onset of a variety of pathologic events, such as coronary heart disease [2], cancer [3], type 2 diabetes [4], and the degenerative diseases associated with aging [5,6].

One of the hallmarks of neurodegeneration is the loss of certain neurons. ROS can contribute to neuronal cell death by either apoptosis or necrosis, and mitochondria are viewed as one of the most pivotal sensors and amplifiers of cell death [7]. Mitochondrial outer membrane permeabilization results in the release of multiple proteins, such as cytochrome *c* and endonuclease G, into the cytosol. This leads to caspase activation in the cytosol, disruption of the mitochondrial respiratory chain, loss of mitochondrial transmembrane potential, overproduction of ROS, finally resulting in cell death [8,9,10].

Recently accumulating evidence has indicated that, in addition to mitochondria, lysosomes also play important roles in the cell death signaling induced by ROS [11,12,13]. The initiation of lysosomal membrane permeabilization (LMP) is one of the crucial steps leading to cell death. As a result of the degradation of iron-containing macromolecules, lysosomes can accumulate large amounts of iron, which is known to catalyze Fenton reactions. High levels of ROS attack the membrane lipids, damage the integrity of lysosomal membranes directly, resulting in the release of proteases into the cytosol and the induction of uncontrolled necrotic cell death; in contrast, mild oxidative stress initiates a cascade of events leading to the formation of “pores” on lysosomal membrane, causing partial and selective LMP and inducing apoptotic cell death. Multitudes of parallel pathways involved in ROS-induced LMP make cell death a highly complex process. An important molecule that bridges the LMP and cell death is Bid (BH3 interacting domain death agonist) [14]. Our previous findings indicated that, during the initiation phase of ROS-induced cell death, tBid (truncated Bid) formed by activated caspase 8 is targeted and inserted into the lysosomal membranes by interaction with phosphatidic acid, where tBid changes conformation, forms homooligomers, and triggers the formation of non-bilayer lipid phases, which account for the formation of lipidic pores and the consequent initiation of LMP. As a result of LMP, lysosomal proteases are redistributed into the cytosol, where they culminate in the lysosome-dependent apoptotic signaling [15]. Accordingly, agents that can stabilize the lysosomal membranes might protect the cells from lysosome-dependent cell death.

Various molecules derived from natural antioxidant vitamins or micronutrients show neuroprotection effects *in vitro* and *in vivo*, probably by attenuating ROS-mediated cell death [16,17]; however, whether they prevent cell death by stabilizing lysosomes, and, if yes, what the underlying mechanisms are remain elusive. In this study, we investigated the effects of a vitamin E derivative, γ-l-glutamyl-*S*-[2-[[[3,4-dihydro-2,5,7,8-tetramethyl-2-(4,8,12-trimethyltridecyl)-2*H*-1-benzopyran-6-yl]oxy]carbonyl]-3-[[2-(1*H*-indol-3-yl)ethyl]amino]-3-oxopropyl]-l-cysteinylglycine sodium salt (ESeroS-GS), on ROS-induced neuronal cell death. The molecular mechanisms by which ESeroS-GS stabilizes lysosomes were also explored.

## 2. Results and Discussion

### 2.1. ESeroS-GS Scavenged ABTS^•+^ and DPPH Free Radicals

**Figure 1:**
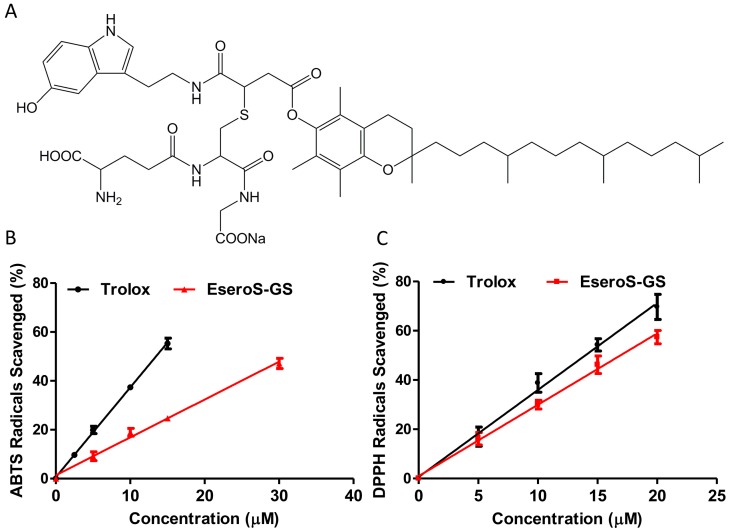
Scavenging effects of ESeroS-GS and Trolox on free radicals. (**A**) structure of ESeroS-GS; (**B**) scavenging effects of ESeroS-GS and Trolox on ABTS^•+^ free radicals; (**C**) scavenging effects of ESeroS-GS and Trolox on DPPH free radicals.

γ-l-glutamyl-*S*-[2-[[[3,4-dihydro-2,5,7,8-tetramethyl-2-(4,8,12-trimethyltridecyl)-2*H*-1-benzo-pyran-6-yl]oxy]carbonyl]-3-[[2-(1*H*-indol-3-yl)ethyl]amino]-3-oxopropyl]-l-cysteinylglycine sodium salt (ESeroS-GS; Figure 1A) is a water-soluble derivative of α-tocopherol (vitamin E). We reported previously that ESeroS-GS can act as an anti-inflammatory agent that inhibits the activation of astrocytes [18] or macrophages [19] by modulating NF-κB signaling via a lipid raft-dependent mechanism. We also found that ESeroS-GS induced cell death in different breast cancer cells but showed no significant effects on MCF-10A mammary epithelial cells [20]. However, the potential antioxidant properties of ESeroS-GS remain elusive. Taking the multifactorial character of oxidative stress into account, we decided to evaluate its *in vitro* free radical scavenging abilities using two different assays.

Firstly, we determined the scavenging activities of ESeroS-GS against the hydrophilic cation radical of 2,2’-azinobis(3-ethylbenzothiazoline)-6-sulfonate (ABTS^•+^) by measuring the decolorization of the ABTS^•+^ radicals at 734 nm [21,22]. 6-Hydroxy-2,5,7,8-tetramethy-chroman-2-carboxylic acid (Trolox), a water-soluble analog of α-tocopherol, was used as the reference compound. The extent of scavenging of the ABTS^•+^ was plotted as a function of antioxidant concentration, as shown in Figure 1B. Both ESeroS-GS and Trolox scavenged ABTS^•+^ free radicals dose-dependently. The IC_50_ values for ESeroS-GS and Trolox in scavenging ABTS^•+^ radicals were 31.4 and 13.5 μM, respectively.

Then, we determined the scavenging activities of ESeroS-GS and Trolox against hydrophobic 2,2-diphenyl-1-picrylhydrazyl (DPPH) stable free radicals [23,24]. ESeroS-GS decreased the signal of DPPH radicals in a concentration-dependent manner, as shown in Figure 1C. The IC_50_ values for ESeroS-GS and Trolox in scavenging DPPH radicals were 16.9 and 14.3 μM, respectively.

### 2.2. ESeroS-GS Protected Neuronal Cells from Oxidative Stress

**Figure 2:**
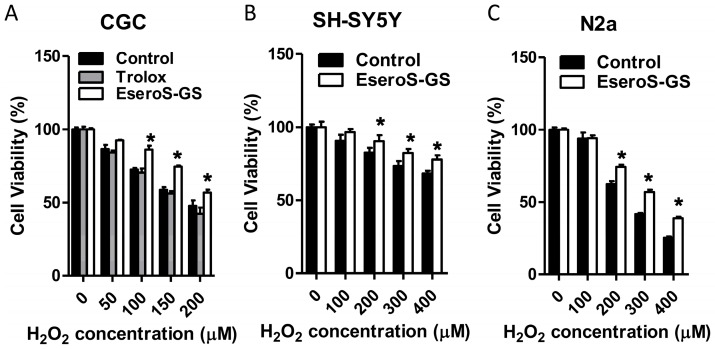
Protective effects of ESeroS-GS on neuronal cells. (**A**) protective effects of ESeroS-GS on cerebellar granule cells; (**B**) protective effects of ESeroS-GS on human SH-SY5Y neuroblastoma cells; (**C**) protective effects of ESeroS-GS on murine N2a neuroblastoma cells. *: *p* < 0.05 in comparison with control cells.

Since both ESeroS-GS and Trolox scavenged free radicals effectively, we then tested their potential protect effects on neuronal cells exposed to oxidative stress. Primary cultures of cerebellar granule cells, a relatively homogenous population of neurons, were used as the cell model. The viability of neuronal cells was assessed by the 3-(4,5-dimethyl-2-thiazolyl)-2,5-diphenyl-2-H-tetrazolium bromide (MTT) assay, which is based on the reduction of MTT by mitochondrial dehydrogenases. As shown in Figure 2A, treatment with 100 μM H_2_O_2_ decreased the cell viability to 72.5%. In cerebellar granule cells pretreated with 50 μM of ESeroS-GS, treatment with 100 μM H_2_O_2_ decreased the cell viability to 86.3%, suggesting that ESeroS-GS attenuated H_2_O_2_-induced cell death effectively. However, Trolox did not show apparent protective effects on neuronal cells, even at higher concentrations (100 μM).

The protective effects of ESeroS-GS against oxidative stress-induced cell death were further evaluated with two immortalized cell lines, the human SH-SY5Y and the murine N2a neuroblastoma cells. ESeroS-GS pretreatment also attenuated H_2_O_2_-induced cell death in these two cell lines, as shown in Figure 2B,C.

### 2.3. Relocation of Lysosomal Chymotrypsin Mediated Cell Death

Although Trolox is more effective than ESeroS-GS in scavenging ABTS^•+^ and DPPH free radicals, it showed no apparent protective effects on H_2_O_2_-induced neuronal cell death. We then hypothesized that ESeroS-GS might protect the neuronal cells not only by scavenging radicals directly, but also via other mechanisms. Lysosomes are one of the pivotal targets of oxidative stress within cells [13,25]. To investigate whether lysosomes are involved in H_2_O_2_-induced neuronal cell death, we measured the permeability of lysosomal membranes after H_2_O_2_ exposure by using acridine orange (AO) as the lysosomotropic fluorophore. AO is a cell-permeable dye that gives rise to red fluorescence at high concentrations and green fluorescence at low concentrations. AO accumulates by proton trapping in intact lysosomes due to the fact that it becomes positively charged in the acidic lysosomal milieu. Following lysosomal membrane permeabilization, AO is released from lysosomes into the cytosol where it emits enhanced green fluorescence that can be monitored by flow cytometry [15].

**Figure 3:**
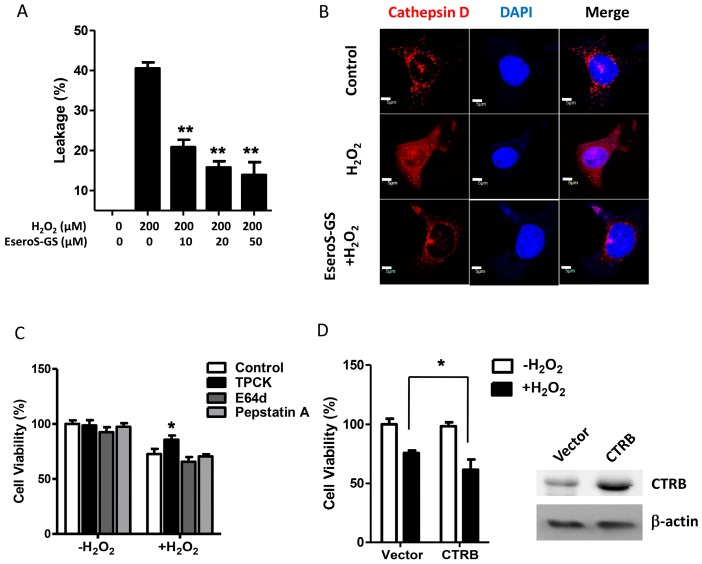
ESeroS-GS inhibits oxidative stress-induced lysosome membrane permeabilization and blocks the relocation of lysosomal chymotrypsin in SH-SY5Y neuroblastoma cells. (**A**) ESeroS-GS inhibits oxidative stress-induced lysosome membrane permeabilization; (**B**) ESeroS-GS blocks the relocation of lysosomal protease. Scale bar: 5 μm; (**C**) inhibition of chymotrypsin prevents oxidative stress-induced cell death; (**D**) Overexpression of chymotrypsin potentiates oxidative stress-induced cell death. The efficacy of chymotrypsin overexpression was evaluated by immunoblot analysis. Transfection with chymotrypsin B (CTRB) vector resulted in a marked (~5 times) increase in cellular chymotrypsin levels in SH-SY5Y neuroblastoma cells. *: *p* < 0.05; **: *p* < 0.01.

The results of flow cytometry indicated that, as a result of oxidative stress, over 40% of SH-SY5Y cells underwent lysosomal membrane permeabilization, as evidenced by the decreased red fluorescence (lysosomal AO) and the increased green fluorescence (cytosolic AO). Pretreatment with ESeroS-GS attenuated the permeabilization of lysosomal membrane effectively in a concentration-dependent manner. In SH-SY5Y cells pretreated with 50 µM of ESeroS-GS, the percentage of cells with permeabilized lysosomes decreased to 14.1 % (Figure 3A).

To further confirm that oxidative stress damaged the lysosomal integrity and triggered the redistribution of lysosomal proteases, we observed the translocation of lysosomal protease cathepsin D, a marker lysosomal protease, in oxidative stressed SH-SY5Y cells by immunofluorescence microscopy. In control cells, cathepsin D (red) was cached in the lysosomes and showed a punctuate pattern (Figure 3B). Exposure of cells to oxidative stress caused the redistribution of cathepsin D into the cytosol, where they showed a diffused pattern. With the ESeroS-GS pretreatment, the redistribution of cathepsin D into the cytosol was attenuated markedly, suggesting that ESeroS-GS prevented the lysosomal membrane from permeabilization and blocked the relocation of lysosomal proteases.

Upon lysosomal membrane permeabilization, dozens of lysosomal proteases were relocated into the cytosol, where they cleaved target molecules and executed the lysosome-dependent cell death. To ascertain which lysosomal protease(s) was (were) responsible for the oxidative stress-cell death, we pretreated SH-SY5Y cells with different protease inhibitors. The results shown in Figure 3C indicated that *N*-p-tosyl-l-phenylalanine chloromethyl ketone (TPCK), the specific chymotrypsin inhibitor, attenuated H_2_O_2_-induced cell death in SH-SY5Y cells. However, (2*S*,3*S*)-trans-epoxysuccinyl-l-leucylamido-3-methylbutane ethyl ester (E-64d) and pepstatin A, two inhibitors of the lysosomal cathepsin families, had no apparent effect on cell death. 

We further manipulated the expression levels of chymotrypsin to ascertain the involvement of chymotrypsin in cell death. Overexpression of chymotrypsin sensitized SH-SY5Y cells to oxidative stress, as shown in Figure 3D. Upon H_2_O_2_ exposure, the viability of SH-SY5Y transfected with chymotrypsin expression vector was 61.5%, significantly lower than that of control cells. These data were consistent with the partial reduction of H_2_O_2_-induced cell death by the chymotrypsin inhibitor TPCK, and clearly indicated that lysosomal chymotrypsin play crucial roles in H_2_O_2_-induced neuronal cell death.

### 2.4. ESeroS-GS Stabilized Lysosomal Membranes

The above data suggested that pretreating neuronal cells with ESeroS-GS effectively decreased the permeabilization of lysosomal membranes, attenuated the redistribution of lysosomal proteases into the cytosol, and prevented neuronal cells from cell death. We then asked by which mechanisms ESeroS-GS stabilize the lysosomal membranes. Our previous data indicated that tBid is responsible for the induction of lysosomal membrane permeabilization during cell death [15]. We then observed the effects of ESeroS-GS on tBid-mediated membrane permeabilization in a relatively simple context by using isolated lysosomes and liposomes that mimick the lipid composition of lysosomes.

**Figure 4:**
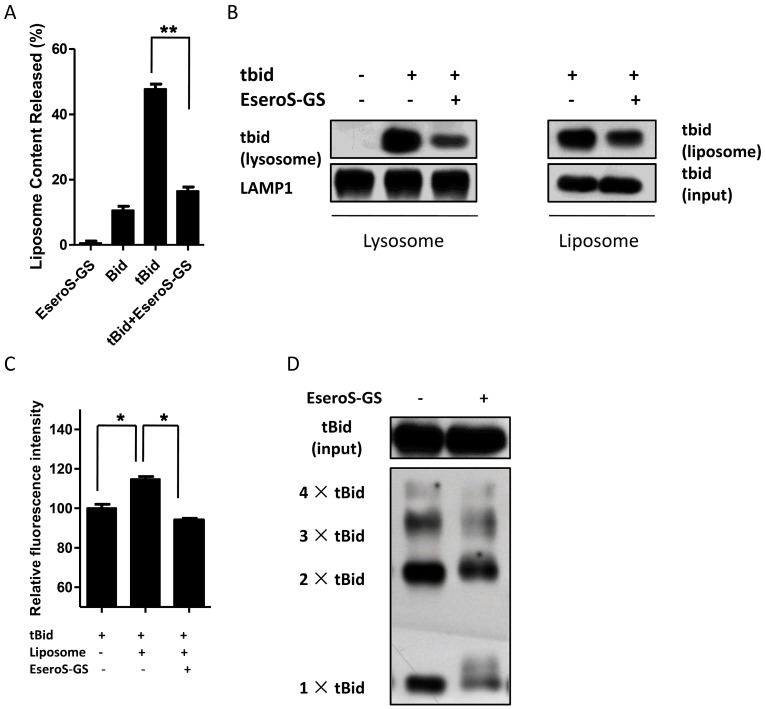
ESeroS-GS stabilizes lysosomal membranes by inhibiting the insertion and oligomerization of tBid to membranes. (**A**) ESeroS-GS inhibits tBid-induced permeabilization of model membranes; (**B**) ESeroS-GS attenuates the translocation of tBid to lysosomes and model membranes; (**C**) ESeroS-GS decreases the interaction between tBid and model membranes; (**D**) ESeroS-GS blocks the oligomerization of tBid within the membrane circumstances. *: *p* < 0.05; **: *p* < 0.01.

We first measured the effects of ESeroS-GS on tBid-mediated permeabilization of model membranes (large unilamellar vesicles; LUVs). As shown in Figure 4A, tBid induced the leakage of LUVs, mimicking the phospholipid composition of lysosomal membranes significantly. In LUVs incubated with tBid, up to 47.5% of the contents encapsulated within the LUVs were released as a result of membrane permeabilization. The addition of ESeroS-GS attenuated the tBid-induced membrane permeabilization effectively, and only 16.8% of the LUV contents were released. 

Our previous data indicated that the translocation of tBid to lysosomes is the initial key step in the induction of lysosomal membrane permeabilization; we then measured the impact of ESeroS-GS on tBid translocation to lysosomes in a cell-free system. Lysosomes were isolated from rat cerebral cortex and then were incubated with tBid. As shown in Figure 4B, incubation of tBid with lysosomes caused its significant translocation to the lysosomal membranes, and the addition of ESeroS-GS could decrease translocation of tBid to the lysosomal membranes.

The impact of ESeroS-GS on tBid translocation was also investigated with model membrane systems. tBid translocated to the phosphatidic acid-containing LUVs rapidly, as shown in Figure 4B, and this translocation could be partially abolished by ESeroS-GS, similar to the results observed in isolated lysosomes.

We also determined the effects of ESeroS-GS on the interaction between tBid and lipid bilayer by fluorescence spectrometry. *N*-(1-pyrenyl)maleimide (PM) is an environmentally sensitive fluorescent probe, whose fluorescence intensity increases obviously in a hydrophobic environment. The fluorescence intensity of tBid labeled with PM was increased after incubating with liposomes, suggesting the insertion of tBid into the lipid bilayer. However, the addition of ESeroS-GS decreased the PM fluorescence intensity significantly, suggesting that the insertion of tBid into the lipid bilayer was blocked by ESeroS-GS (Figure 4C).

After the insertion of tBid molecules into the membrane, they formed homooligomers, which were the structural basis of the lipidic pore that was responsible for the membrane permeabilization. We further investigated the effects of ESeroS-GS on tBid oligomerization in model membranes, mimicking the phospholipid composition of lysosomal membranes. tBid was incubated with liposomes followed by centrifugation to separate the membranous fraction from the soluble fraction. Both fractions were treated with the bis[2-(sulfosuccinimidooxycarbonyloxy)ethyl] sulfone (sulfo-BSOCOES) cross-linker and analyzed by immunoblotting. After adding tBid to liposomes, a substantial amount of tBid was incorporated into the liposomal membrane, with the appearance of 14-kDa (monomer), 28-kDa (dimer), 42-kDa (trimer), 56-kDa (tetramer) and higher molecular weight band (homooligomers) cross-linked complexes. Significantly less tBid oligomerization was incorporated into the liposomal membrane when EseroS-GS was added into the liposomes, suggesting that ESeroS-GS attenuated the oligomerization of tBid.

## 3. Materials and Methods 

### 3.1. Chemicals and Antibodies

γ-l-Glutamyl-*S*-[2-[[[3,4-dihydro-2,5,7,8-tetramethyl-2-(4,8,12-trimethyltridecyl)-2*H*-1-benzo-pyran-6-yl]oxy]carbonyl]-3-[[2-(1*H*-indol-3-yl)ethyl]amino]-3-oxopropyl]-l-cysteinylglycine sodium salt (ESeroS-GS) was a generous gift from Kazumi Ogata of Senju Pharmaceutical Co. Ltd. (Osaka, Japan). 6-Hydroxy-2,5,7,8-tetramethychroman-2-carboxylic acid (Trolox), 2,2’-azinobis (3-ethylbenzothiozoline-6-sulphonic acid) diammonium salt (ABTS), 1,1-dipheny-2-picrylhydrazyl (DPPH), 5-carboxyfluorescein (CF), *N*-p-tosyl-l-phenylalanine chloromethyl ketone (TPCK), (2*S*,3*S*)-trans-epoxysuccinyl-l-leucylamido-3-methylbutane ethyl ester (E-64d), pepstatin A, 2-[4-(2-Hydroxyethyl)-1-piperazinyl]ethanesulfonic acid (HEPES), 3-(4,5-dimethyl-2-thiazolyl)-2,5-diphenyl-2-H-tetrazolium bromide (MTT) and ethylenediaminetetraacetic acid disodium salt (EDTA) were from Sigma-Aldrich (St Louis, MO, USA). 1,2-Dioleoyl-sn-glycero-3-phosphate (DOPA), 1,2-dioleoyl-sn-glycero-3-phosphocholine (DOPC) and 1,2-dioleoyl-sn-glycero-3-phosphoethanolamine (DOPE) were from Avanti Polar Lipids (Alabaster, AL, USA). Acridine orange (AO), *N*-(1-pyrenyl)maleimide (PM), 4’,6-diamidino-2-phenylindole (DAPI), cell culture medium, cell culture supplements and fetal bovine serum were from Life Technologies (Eugene, OR, USA). Primary antibodies against cathepsin D and Bid were from Santa Cruz Biotechnology (Santa Cruz, CA, USA). Horseradish peroxidase (HRP)-labeled and 6-tetramethylrhodamine isothiocyanate (TRITC)-labeled second antibodies were from Sigma-Aldrich. Other reagents made in China were of analytical grade.

### 3.2. Expression and Labeling of Recombinant Proteins 

Recombinant full-length Bid and caspase 8 were obtained as we described previously [26]. Truncated Bid (tBid; includes N-terminal fragment 1–59 and C-terminal fragment 60–195) was obtained by adding caspase 8 to the full-length Bid, and then incubating overnight at 37 °C [27]. In some experiments, tBid was labeled with fluorescent probe *N*-(1-pyremyl)maleimide (PM). Before labeling, Bid was dialyzed extensively against Tris buffer (20 mM Tris-HCl, pH 7.5, 300 mM NaCl) and then labeled with PM dye (3 mol of PM to 1 mol of tBid) in the same buffer with rotation at 30 °C for 2 h in the dark. After the reaction, the protein sample was dialyzed against Tris buffer to remove the excessive PM dye.

### 3.3. Free Radical Scavenging Assays

The scavenging effect of ESeroS-GS on the hydrophilic ABTS free radicals (ABTS^•+^) was measured by the decolorization of ABTS^•+^ at 734 nm [21,22]. ABTS dissolved in water at a 7 mM concentration was reacted with 2.45 mM potassium persulfate in the dark at room temperature for 12–16 h. Then, the ABTS^•+^ solution was diluted with 150 mM phosphate buffer (pH 7.3) to an absorbance of 0.700 ± 0.020 at 734 nm and equilibrated at 30 °C. After addition of 1.0 ml of diluted ABTS^•+^ solution (A_734nm_ = 0.700 ± 0.020) to 10 μL of ESeroS-GS or Trolox solutions (final concentration 0~15 μM), the absorbance reading at 734 nm was taken exactly 5 min after initial mixing. The percentage scavenging of ABTS^•+^ was calculated by:

I(%) = [(Ao − Ax)/Ao] × 100(1)

Here, Ax and Ao were the absorbance at 734 nm of samples with and without antioxidants, respectively.

The scavenging of hydrophobic DPPH free radicals by ESeroS-GS was examined by the Electron Spin Resonance (ESR) technique [23,24]. DPPH was dissolved in ethanol to give a 100 μM solution and mixed with equal volumes of ESeroS-GS or Trolox solutions, then transferred to quartz capillary which was inserted into the cavity of the Bruker ER200SRC ESR spectrometer (Bruker, Karlsruhe, Germany). ESR spectra were recorded exactly 2 min after initial mixing. ESR measurement conditions were as follows: central field 3475 G, modulation frequency 100 kHz, modulation amplitude 2 G, microwave power 10 mW, scan width 200 G, gain 6.3 × 10^5^, temperature 298 K. The scavenging effects on DPPH free radical were calculated by:

I(%) = [(Ho − Hx)/Ho] × 100(2)

Here, Hx and Ho were the ESR signal intensities of samples with and without antioxidants, respectively.

### 3.4. Cell Culture and H_2_O_2_ Treatment

Primary cultures of rat cerebellar granule cells were prepared from postnatal day 6Sprague-Dawley rat pups (Vital River Experimental Animal Center, Beijing, China), with the regulations approved by the Institutional Animal Care and Use Committee of Institute of Biophysics, Chinese Academy of Sciences (IACUC-IBP) [28]. Cells were plated on 96-well multidishes previously coated with poly-l-lysine. Culture medium consisted of Dulbecco’s modified Eagle medium (DMEM) supplemented with KCl (19.6 mM), glutamine (2 mM), HEPES (10 mM) and fetal bovine serum (10%, *v*/*v*). Mouse neuroblastoma N2a cells and human neuroblastoma SH-SY5Y cells were obtained from the American Type Culture Collection (ATCC; Manassas, VA, USA) and were cultured under conditions recommended by ATCC. Cerebellar granule cells, N2a cells and SH-SY5Y cells were exposed to oxidative stress by incubation with H_2_O_2_ in serum-free cell culture medium for 30 min and then cultured in fresh medium containing serum for 12 h. In some experiments, cells were pretreated with ESeroS-GS, Trolox, TPCK, E-64d or pepstatin for 1 h before H_2_O_2_ exposure.

### 3.5. Transient Overexpression of Chymotrypsin

For the construction of chymotrypsin overexpression vectors, open reading frames encoding chymotrypsinogen were amplified by PCR with appropriate restriction sites and cloned into pcDNA3.1 vector, producing a pcDNA3.1-Ctr vector encoding myc-tagged chymotrypsin [26]. SH-SY5Y cells were transfected with pcDNA3.1-Ctr vector by using Fugene HD reagent (Promega, Madison, WI, USA) according to the manufacturer’s instructions. Then, cells were exposed to 200 μM H_2_O_2_ for 30 min and cultured in fresh medium for 12 h. Immunoblot analysis was performed to evaluate the efficacy of chymotrypsin overexpression.

### 3.6. Assessment of Cell Death

The viability of cells was evaluated by MTT assay, according to methods we described previously [20]. In brief, cells cultured in 96-well multi-dishes were treated with different concentration of H_2_O_2_ for 30 min, and then cultured in fresh medium for 12 h. MTT reagent was added into cells and cells were incubated for additional 1 h. Then, 0.2 mL of lysis solution (10% SDS, 25% *N*,*N*-dimethylformamide, pH 3.5) was added and the optical density at 570 nm was measured by a Thermo Fisher microplate reader (Thermo Fisher, Beijing, China).

### 3.7. Measurement of Lysosomal Membrane Permeabilization

Lysosomal permeabilization was determined in intact cells by staining with acridine orange (AO), a lysosomotropic fluorophore [29,30]. In brief, SH-SY5Y cells were loaded with AO (5 μg/mL) for 30 min and then exposed to 200 μM H_2_O_2_ for 30 min. AO-emitted red (lysosomal) and green (nuclear and cytosolic) fluorescence were analyzed with a flow cytometer (FACSCalibur; BD, Mountain View, CA, USA). Cells with enhanced green fluorescence and decreased red fluorescence were regarded as lysosomal membrane-permeabilized cells.

The permeabilization of lysosomes was further confirmed by the relocation of lysosomal cathepsin D. SH-SY5Y cells cultured on glass coverslips were fixed with cold methanol in −20 °C for 20 min and incubated with ice-cold solution containing 95% ethyl alcohol and 1% acetic acid for 5 min. After washing with PBS twice, cells were blocked for 1 h at room temperature with PBS containing 5% goat serum albumin. Cells were incubated with anti-cathepsin D antibody overnight at 4 °C and then incubated with TRITC-labeled secondary antibody at room temperature for 2 h. Nuclei were stained with 4’,6-diamidino-2-phenylindole (DAPI). Cells were visualized by Z-Stack imaging with a confocal microscope (Olympus FV1000; Tokyo, Japan) and processed using Fluoview software (Version 2.0, Olympus).

### 3.8. In Vitro Measurement of Membrane Permeabilization

Large unilamellar vesicles (LUVs) mimicking the composition of lysosomal membranes were used as model systems to investigate the impact of ESeroS-GS on membrane permeabilization [15]. In brief, DOPC (70%), DOPE (20%) and DOPA (10%) were mixed from stocks dissolved in chloroform. The organic solvent was removed by evaporation under a stream of nitrogen gas, followed by incubation for 2 h in a vacuum to ensure complete solvent removal. Lipid films were resuspended in HEPES buffer (10 mM HEPES, pH 7.4, 50 mM NaCl, 0.2 mM EDTA), and subjected to 10 freeze-thaw cycles. Large unilamellar vesicles (LUVs) were then formed by extrusion through 100-nm nucleopore polycarbonate membranes.

The permeabilization of membrane was determined by monitoring the fluorescence of 5-carboxyfluorescein (CF)-containing liposomes. To prepare CF-containing liposomes, 50 mM CF was dissolved in the HEPES buffer, and LUVs were prepared with the CF-containing HEPES buffer. Non-entrapped fluorophores were removed by centrifugation at 45,000 rpm for 30 min with a Beckman TL-100.3 ultracentrifuge (Beckman, Brea, CA, USA). The leakage assay was performed as described previously [31]. Following incubation of tBid with LUVs (phospholipids concentration 100 μM) with or without EseroS-GS in 100 μL of HEPES buffer at 30 °C for 30 min, the release of CF was quantified using a Hitachi F-4500 flurospectrometer (Hitachi, Tokyo, Japan) with 5 nm bandwidths centered at 493 and 513 nm for excitation and emission, respectively (R). The control followed the same procedure except for the addition of buffer instead of protein (B). Addition of Triton X-100 to a final concentration of 1% (*v*/*v*) was used as a positive control for 100% CF release (T). The percentage of leakage was determined by:

Leakage(%) = [(R − B)/(T − B)] × 100(3)

### 3.9. Binding of tBid to Lysosomal and Model Membranes

Lysosomes were purified from rat cerebral cortex and were suspended in buffer containing 250 mM sucrose, 20 mM HEPES, pH 7.2 [26]. Then, lysosomes were incubated with tBid with or without EseroS-GS for 30 min, respectively, followed by centrifugation at 18,000× *g* for 10 min. The pellets (lysosomes) were subjected to immunoblot analysis to detect the binding of tBid to lysosomal membranes.

For detection of tBid bound to model membranes, LUVs prepared from DOPC (70%), DOPE (20%) and DOPA (10%) was incubated with tBid with or without ESeroS-GS in 100 μL of HEPES buffer at 30 °C for 30 min. Then, the samples were centrifuged at 45,000 rpm for 30 min. The pellets were washed three times with the HEPES buffer and then were subjected to immunoblot analysis to detect the binding of tBid to LUV membranes.

The insertion of tBid into the LUV bilayer membranes was also analyzed by measuring the fluorescence of PM-labeled tBid. Briefly, tBid was labeled with PM to form PM-labeled tBid and then incubated with LUVs prepared from DOPC (70%), DOPE (20%) and DOPA (10%) in HEPES at 30 °C for 30 min. Fluorescence measurements were performed with a Hitachi F-4500 fluorescence spectrometer set to 340 nm excitation and 376 nm emission with 5 nm slit widths.

### 3.10. Assay of tBid Oligomerization

The oligomerization of tBid was measured by a cross-linking assay. tBid was incubated with LUVs prepared from DOPC (70%), DOPE (20%) and DOPA (10%) in buffer containing 10 mM HEPES (PH 7.4), 0.1M NaCl at 37 °C for 30 min and then the samples were centrifuged at 45,000 rpm for 30 min. Sulfo-BSOCOES (in dimethyl sulfoxide) was added to the pellets from at a final concentration of 10 mM. After incubation for 2 h at room temperature, the cross-linker was quenched by the addition of 1 M Tris-HCl (pH 7.5) to a final concentration of 20 mM. Then, the samples were lysed and subjected to SDS-PAGE. Immunoblot analysis was performed to analyze the oligomerization of tBid.

### 3.11. Immunoblot Analysis

Samples were resolved by 15% SDS-PAGE, transferred to polyvinylidene fluoride membranes, and probed with proper primary antibodies. The membranes were incubated with peroxidase-conjugated secondary antibodies and visualized using a Amersham Pharmacia ECL chemiluminescent kit (GE, Marlborough, MA, USA) and Kodak X-OMAT films (Rochester, NY, USA).

### 3.12. Data Analysis

All data were expressed as the mean ± SD unless otherwise indicated. Differences between groups were compared by analysis of variance followed by *post hoc* Bonferroni tests to correct for multiple comparisons. Differences were considered to be statistically significant at *p* < 0.05 (*) or *p* < 0.01 (**).

## 4. Conclusions

We reported here that ESeroS-GS effectively scavenged free radicals and protected neuronal cells from oxidative stress-induced cell death by stabilizing lysosomes. Oxidative stress induced lysosomal membrane permeabilization rapidly and caused the redistribution of lysosomal proteases, which was responsible for the neuronal cell death. ESeroS-GS abolished the interaction between tBid and the lysosomal membranes, blocked the translocation of tBid to the lysosomal membranes, decreased its oligomerization within the membrane circumstances, prevented the lysosomal membrane permeabilization, and thus attenuated the neuronal cell death. These data suggest that ESeroS-GS protected the neuronal cells from oxidative stress by stabilizing lysosomal membranes, and thus might act as a novel neuroprotector for neuronal diseases associated with oxidative stress. However, other mechanisms, such as the induction and activation of Nrf2/Keap1/ARE pathway, may also be involved in the neuroprotection by ESeroS-GS. We will focus on this key pathway in future research.

## Abbreviations

The following abbreviations are used in this manuscript:

EseroS-GS	γ- l-glutamyl-*S*-[2-[[[3,4-dihydro-2,5,7,8-tetramethyl-2-(4,8,12-trimethyltridecyl)-2*H*-1-benzopyran-6-yl]oxy]carbonyl]-3-[[2-(1*H*-indol-3-yl)ethyl]amino]-3-oxopropyl]-l-cysteinylglycine sodium salt
ROS	reactive oxygen species
LMP	lysosomal membrane permeabilization
Bid	BH3 interacting domain death agonist
tBid	truncated Bid
ABTS·^+^	cation radical of 2,2’-azinobis(3-ethylbenzothiazoline)-6-sulfonate
DPPH	2,2-diphenyl-1-picrylhydrazyl free radical
ESR	Electron Spin Resonance
Trolox	6-hydroxy-2,5,7,8-tetramethychroman-2-carboxylic acid
MTT	3-(4,5-dimethyl-2-thiazolyl)-2,5-diphenyl-2-*H*-tetrazolium bromide
AO	acridine orange
TPCK	*N*-p-tosyl-l-phenylalanine chloromethyl ketone
E64d	(2*S*,3*S*)-trans-epoxysuccinyl-L-leucylamido-3-methylbutane ethyl ester
LUV	large unilamellar vesicle
PM	*N*-(1-pyrenyl)maleimide
DOPA	1,2-dioleoyl-sn-glycero-3-phosphate
DOPC	1,2-dioleoyl-sn-glycero-3-phosphocholine
DOPE	1,2-dioleoyl-sn-glycero-3-phosphoethanolamine
HRP	horseradish peroxidase
TRITC	6-tetramethylrhodamine isothiocyanate
CF	5-carboxyfluorescein
sulfo-BSOCOES	bis[2-(sulfosuccinimidooxycarbonyloxy)ethyl] sulfone
EDTA	ethylenediaminetetraacetic acid disodium salt
